# Redefinition of the genus *Allonychiurus* Yoshii, 1995 (Collembola, Onychiuridae) with description of a new species from China

**DOI:** 10.3897/zookeys.78.977

**Published:** 2011-01-28

**Authors:** Sun Xin, Chen Jian-Xiu, Deharveng Louis

**Affiliations:** 1 Department of Biological Science and Technology, Nanjing University, Nanjing 210093, P.R. China; 2 Muséum national d’Histoire naturelle, UMR7205 du CNRS, CP50, 45, rue Buffon, 75005 Paris, France

**Keywords:** Collembola, *Allonychiurus*, *Micronychiurus*, *Spinonychiurus*, *Thalassaphorura*, *Thibaudichiurus*, chaetotaxy, taxonomy, China

## Abstract

In this paper, we describe a new species of the genus Allonychiurus Yoshii, 1995, characterized by the presence of an apical swelling on the fourth antennal segment as well as a combination of chaetotaxic and pseudocellar characters. The genus Allonychiurus is redefined. Four of its species are considered as *incertae sedis*: Allonychiurus michelbacheri (Bagnall, 1948), Allonychiurus spinosus (Bagnall, 1949), Allonychiurus caprariae (Dallai, 1969) and Allonychiurus sensitivus (Handschin, 1928). The three species Allonychiurus borensis (Beruete, Arbea & Jordana, 1994), Allonychiurus sensilatus (Thibaud & Massoud, 1979) and Allonychiurus vandeli (Cassagnau, 1960) are removed from Allonychiurus and placed in Micronychiurus Bagnall, 1949, Thalassaphorura Bagnall, 1949 and Spinonychiurus Weiner, 1996 respectively. The synonymy of Thibaudichiurus Weiner, 1996 with Allonychiurus is rejected and Allonychiurus foliatus (Rusek, 1967) and Allonychiurus mariangeae (Thibaud & Lee, 1994) are re-allocated to Thibaudichiurus. List and identification key to the world species of the genus are given.

## Introduction

The genus Allonychiurus Yoshii, 1995 includes 23 species according to Bellinger et al. (2010). They are distributed in Asia, Europe and America. However, as stressed by Sun et al. (2009), generic assignment of most of these species is disputable. To improve this confusing situation, we re-examined all available taxonomic descriptions and type specimens of several species. We redefined the genus accordingly. As a result, five species are translocated to other genera and four are considered as *incertae sedis*. A new species discovered in China, Allonychiurus antennalis sp. n., is described.

## Redefinition of Allonychiurus Yoshii, 1995

**Type-species: **Onychiurus flavescens** Kinoshita, 1916: p. 458**

### Diagnosis

Onychiurinae Thalassaphorurini. Postantennal organ oval, with numerous compound vesicles perpendicular to the long axis; antennal basis rather well delimited. Clubs of antenna III organ smooth or granulated. No invaginated apical bulb on Ant. IV. Labral chaetae formula 4/3,4,2; labium of AC type (*sensu* Fjellberg 1999); chaeta d0 present on head. No multiplication of dorsal pseudocelli; 3 (rarely 2) anterior pseudocelli on head, located inside the area antennalis; Th. I tergite usually with 1 or 2 pseudocelli per half-tergite (rarely absent). Abd. III sternite not subdivided into two subsegments; 4 (rarely 2, 3 or 5) pseudocelli per half-tergite on Abd. IV; 3 (rarely 2 or 4) pseudocelli per half-tergite on Abd. V (2–3 postero-internal and 0–1 postero-lateral). Abd. VI with 1 or 2 uneven axial chaetae (a0 or p0, or both); anal spines present. Tibiotarsus with 9 or 11 chaetae in the distal whorl, clavate tenent hairs absent. Furcal rudiment as finely granulated area with 4 posterior minute dental chaetae regularly arranged in 2 rows. Two or three rows of manubrial chaetae posteriorly to the 4 dental chaetae.

## Discussion

The genus Allonychiurus is very similar to Onychiurus Gervais, 1841 differing from it by a furcal area with 4 small posterior chaetae arranged in two rows versus arranged in one row. It is also the only difference between Thalassaphorurini and Onychiurini. The attempt to restrict the genus Allonychiurus to species with 11 distal chaetae on tibiotarsus (Sun et al. 2009) versus 9 in Onychiurus cannot be retained on current available evidence, as discussed below.

### The problem of species assigned to Allonychiurus

The genus Allonychiurus was described by Yoshii (1995) as a subgenus of Onychiurus Gervais, 1841, to include species of the *flavescens*-group of Paronychiurus previously recognized by Weiner (1989). This last author upgraded it to genus level in 1996, characterizing it mostly on the basis of its furcal area similar to that of Thalassaphorura Bagnall, 1949, and its post-antennal organ with several compound vesicles. Recently, Sun et al. (2009) restricted the definition of Allonychiurus to species with 11 chaetae in the distal whorl of tibiotarsus (a character erroneously stated as being drawn from the Weiner 1996 diagnosis); according to this conception, only four species (out of the 24 species listed at this time on the Janssens and Christiansen website at http://www.collembola.org) could be confirmed as Allonychiurus (*flavescens*, *jongaksanensis*, *shanghaiensis* and *shinbugensis*), in addition to Allonychiurus megasomus that Sun et al. (2009) described in their paper. The authors also reallocated tentatively two species, Allonychiurus edinensis (Bagnall, 1935) and Allonychiurus subedinensis (Arbea & Jordana, 1985) to the genus Spinonychiurus Weiner, 1996, a move formally confirmed by Kaprus’ and Tsalan (2009); they stressed that 12 species did not match the genus as they defined it, nine because they had fewer than 11 chaetae in the distal whorl of tibiotarsus, two because they had smooth clubs in antenna III organ, and one because it had simple PAO vesicles. The remaining 6 species were not documented for distal tibiotarsal chaetae, and their status was considered as doubtful. In short, 80% of species assigned to Allonychiurus did not match the Sun et al. (2009) definition of the genus before our study.

### Thalassaphorurini versus Onychiurini

A further concern is that furcal area chaetotaxy, i.e. the diagnostic character that Pomorski (1998) proposed to distinguish the tribes Thalassaphorurini, where Allonychiurus is placed, and Onychiurini, is not documented in most of the 23 species assigned to Allonychiurus by Bellinger et al. in 2010. These species could belong as well to a genus of Onychiurini, like Onychiurus. In this respect, a few Onychiurus species described in the recent paper of Pomorski, Furgoł and Christiansen (2009) have a furcal area similar to that of Allonychiurus, but the authors do not formally discuss the important taxonomic implications of this finding. They nevertheless recognize that «Many of the features he (Pomorski) used to redefine the genus (Onychiurus) are not given in many descriptions». A large number of species assigned to Allonychiurus and Onychiurus in Bellinger et al. (2010) need therefore to be re-examined in detail, as it is not known if they match the modern diagnoses of these genera. In parallel, differences between both genera as well as between Onychiurini and Thalassaphorurini have to be re-assessed.

### The chaetotaxy of tibiotarsus

The use of the number of chaetae in the distal whorl of tibiotarsus as a diagnostic character to define Allonychiurus deserves further comments. This character is not mentioned in the published descriptions of Allonychiurus by Yoshii (1995) and Weiner (1996). Bellinger et al. (2010) key out the genus as having “more than 7” chaetae in this whorl. Sun et al. (2009) characterize Allonychiurus as having 11 chaetae in the distal whorl of tibiotarsus. Actually, only six species have these 11 chaetae including the type species of the genus, Allonychiurus flavescens (though it needs to be confirmed on type locality specimens) and the new species Allonychiurus antennalis described here. Among the species listed as Allonychiurus in the web site of Janssens and Christiansen at this time, Sun et al. (2009) recognized 9 species with less than 11 distal tibiotarsal chaetae (easily seen on original drawings of Allonychiurus mediasetus (Lee, 1974), and Allonychiurus pseudocellitriadis (Lee, 1974), less obvious for other species). Other morphological characters are very similar between 9- and 11-chaetae species. In order to avoid the splitting of Allonychiurus in weakly defined entities, and given our poor knowledge of other diagnostic characters (furcal area and antenna III organ) in several species, we define Allonychiurus as having 9 or 11 chaetae in the distal whorl of tibiotarsus. This is in line with the other large genus of Thalassaphorurini, i.e. Thalassaphorura, where distal tibiotarsal chaetae are 7 or 9 (Sun et al. 2010), suggesting that this character may have lower taxonomical value in some genera than recently thought.

### Taxonomical approach

In this contribution and as a first step, we address the taxonomic problems raised above in three ways. First, in order to accommodate several species that would otherwise necessitate the creation of new poorly defined genera, we extended the diagnosis of Allonychiurus of Sun et al. (2009) to include species with sensory clubs of antenna III organ smooth or granulated (*versus* only granulated), and species with 9 or 11 distal chaetae on tibiotarsus (*versus* only 11). This new definition is compatible with the characters of Allonychiurus extracted from the key of Bellinger et al. (2010). Second, we remove from the Allonychiurus list of Bellinger et al. (2010) nine species that do not match diagnostic characters of Allonychiurus: four are considered *incertae sedis*, and five are reallocated to other genera. Third, we provisionally keep in Allonychiurus several insufficiently described species listed by Bellinger et al. (2010) that do not conflict with the definition of the genus, but could belong as well to other genera like Onychiurus or Thibaudichiurus Weiner, 1996; their generic assignment will have to be checked from fresh material.

## Critical checklist of the world species of Allonychiurus Yoshii, 1995

In the checklist given below, an asterisk (*) indicates that species assignment requires confirmation.

Allonychiurus flavescens (Kinoshita, 1916) (type species of the genus Allonychiurus by original designation). Originally described in the genus Onychiurus from Japan, later found in Korean caves (Yosii and Lee 1963, Yosii 1966, Weiner 1989) and largely distributed across USA (Muzzio 1984, Christiansen and Bellinger 1998). The different populations of Eastern Asia might however represent closely related geographic species (Weiner 1989, Yoshii 1995). Those of USA exhibit geographical variability according to Christiansen and Bellinger (1998), at a level unusual in other Thalassaphorurini species, and would deserve closer examination.

Allonychiurus donjiensis (Lee & Kim, 1994)*. Described in the genus Onychiurus from South Korea, later placed in Allonychiurus by Babenko (2007, following the tentative key of Onychiurinae on *www.collembola.org*). Morphological similarity with Thibaudichiurus mariangeae (Thibaud & Lee, 1994) was stressed in the original description. Smooth sensory clubs of third antennal segment and four protecting papillae in the sense organ of third antennal segment are shared by the two species. Ecology (coastal habitats) and distribution are also similar. A redescription of the species would probably result in its reallocation to Thibaudichiurus.

Allonychiurus hangchowensis (Stach, 1964). Described in the genus Onychiurus from China (Zhejiang: Hangzhou), later placed in Allonychiurus by Bellinger et al. (2010).

Allonychiurus indicus (Choudhuri & Roy, 1965)*. Described in the genus Onychiurus from India (West Bengale), later placed in Allonychiurus by Bellinger et al. (2010).

Allonychiurus jindoensis (Lee & Kim, 1994)*. Described in the genus Onychiurus from South Korea, later placed in Allonychiurus by Babenko (2007, following the tentative key of Onychiurinae on *www.collembola.org*). Remarks regarding the species affinities of Allonychiurus donjiensis with Thibaudichiurus mariangeae apply here.

Allonychiurus jongaksanensis (Weiner, 1989). Described in the genus Paronychiurus from North Korea, later placed in Allonychiurus by Weiner (1996).

Allonychiurus kimi (Lee, 1973). Described in the genus Onychiurus from South Korea, reported from North Korea by Weiner (1989), later placed in Allonychiurus by Yoshii (1995).

Allonychiurus mediasetus (Lee, 1974). Described as Onychiurus mediaseta from South Korea, reported from North Korea by Weiner (1989), later placed in Allonychiurus as Allonychiurus mediaseta by Weiner (1996).

Allonychiurus megasomus Sun, Yan & Chen, 2009. Described from China (Nanjing).

Allonychiurus pamirensis (Martynova, 1975)*. Described in the genus Onychiurus from Tajikistan at high altitude (East Pamir), later placed in Allonychiurus by Bellinger et al. (2010).

Allonychiurus pseudocellitriadis (Lee, 1974). Described in the genus Onychiurus from South Korea, later placed in Allonychiurus by Weiner (1996).

Allonychiurus shanghaiensis (Rusek, 1971)*. Described in the genus Onychiurus from China (Shanghai), later placed in the genus Allonychiurus by Sun et al. (2009).

Allonychiurus shinbugensis (Lee, 1974). Described in the genus Onychiurus from South Korea, reported from North Korea by Weiner (1989), later placed in the genus Allonychiurus by Weiner (1996).

Allonychiurus tianshanicus (Martynova, 1971)*. Described in the genus Onychiurus from Kyrgyzstan at high altitude, later placed in the genus Allonychiurus by Bellinger et al. (2010).

### Incertae sedis

Four of the species currently placed in the genus* Allonychiurus* by Bellinger et al. (2010)are very insufficiently described or exhibit characters that are not those of the genus. They are considered here as Thalassaphorurini *incertae sedis*.

Allonychiurus caprariae (Dallai, 1969). Described in the genus Onychiurus , later placed in Allonychiurus by Bellinger et al. (2010). Only known from the type locality (Capraia Island in Italy). For its antennal basis not differentiated and the presence of 4+4 anterior pseudocelli on head, this species departs from Allonychiurus.

Allonychiurus michelbacheri (Bagnall, 1948). Described in the genus Onychiuroides Bagnall, 1948from the USA, later placed in the genus Allonychiurus by Bellinger et al. (2010). In the original description, considered to be closely related to Onychiurus edinensis Bagnall, 1935 (type of the genus Spinonychiurus Weiner, 1996).

Allonychiurus sensitivus (Handschin, 1928). Described in the genus Onychiurus from Bulgaria** , later placed in Allonychiurus by Bellinger et al. (2010).

Allonychiurus spinosus (Bagnall, 1949). Described in the genus Onychiuroides from Ireland** , later placed in Allonychiurus by Bellinger et al. (2010). The ‘dorsal spines’ of the abdomen mentioned in the original description are likely to be thickened S-chaetae. The “exceptionally long” lateral chaetae on head and body remind a first instar chaetotaxy.

### Species removed from Allonychiurus

Thalassaphorura sensilata (Thibaud & Massoud, 1979), comb. n.

This species was originally described from Lesser Antilles (Central America) in the genus Protaphorura and later transferred to Allonychiurus by Bellinger et al. (2010). PAO with simple vesicles undoubtedly places this species in Thalassaphorura Bagnall, 1949 according to original description, to observation of Sun et al. (2009) and to re-examination of type specimens.

Micronychiurus borensis (Beruete, Arbea & Jordana, 1994), comb. n.

Described in the genus Onychiurus from Spanish Pyrenees, later placed in Allonychiurus by Bellinger et al. (2010). It rather belongs to Micronychiurus Bagnall, 1949 *sensu* Weiner (1996), as indicated by the number of pso on Abd. IV and V (5–7 and 5–6 respectively in Micronychiurus versus a lower number in Allonychiurus). It was correctly placed in the “Onychiurus” *minutus* species-group (equivalent to Micronychiurus) in the original description.

Spinonychiurus vandeli (Cassagnau, 1960), comb. n.

Described in the genus* Onychiurus* from the French Pyrenees at high altitude, later placed in Allonychiurus by Bellinger et al. (2010). We have checked Cassagnau’s type specimens in the Muséum national d’Histoire naturelle of Paris. Because of the absence of d0 on head and the subdivision of Abd. III sternite into two subsegments, the species should be assigned to Spinonychiurus as redefined by Kaprus’ and Tsalan (2009).

Thibaudichiurus foliatus (Rusek, 1967). Described in the genus Onychiurus from China (Shanghai), reallocated to Thibaudichiurus by Weiner (1996), later placed in Allonychiurus by Bellinger et al. (2010). See discussion about the validity of Thibaudichiurus at Thibaudichiurus mariangeae.

Thibaudichiurus mariangeae (Thibaud & Lee, 1994). Described in the genus Onychiurus from South Korea, given as type species of the genus Thibaudichiurus Weiner, 1996, later placed in Allonychiurus by Babenko (2007, following the tentative key of Onychiurinae on *www.collembola.org*), cited again as Thibaudichiurus from Santo island (Vanuatu) by Thibaud (2009). The proposed synonymy of Thibaudichiurus and Allonychiurus has never been documented in the literature, and is not accepted here, after re-examination of the type specimens from the Muséum national d’Histoire naturelle of Paris. The key difference between Thibaudichiurus and Allonychiurus according to Weiner (1996) is the presence of 2+2 *versus* l+1 pseudocelli on first thoracic tergite. The same character is supposed to separate two other genera of Thalassaphorurini (Jailolaphorura Yosii & Suhardjono, 1992 and Thalassaphorura Bagnall, 1949), but was not considered of generic value by Sun Xin et al. (2010), as typical and closely related species of Thalassaphorura may have either 1+1 or 2+2 on this tergite. In the same way, we did not retained this character as discriminant for Thibaudichiurus . However, Thibaudichiurus is maintained here on the basis of its single row of manubrial chaetae posterior to dental chaetae (several in Allonychiurus) (Weiner 1996 and comm. pers.), and the presence of characteristic, thickened chaetae on the male genital plate that are not recorded in Allonychiurus species. According to Pomorski (1998) Thibaudichiurus is also closely related to Tantulonychiurus Pomorski, 1996 from which is differs by modified chaetae of male restricted to the genital plate and position of dorso-median pseudoscelli on abdomen IV and V tergites; we confirm also the difference suspected by Pomorski in number of distal chaetae of tibiotarsi (11 in Thibaudichiurus, 7 in Tantulonychiurus). Arrangement of chaetae on and around furcal area (but not their morphology) is identical between the two genera.

### Note on species ecology and distribution

Allonychiurus occurs in a wide range of habitats. Most described species live in soil and litter of lowland areas (Weiner 1989). The type species of the genus (Allonychiurus flavescens) has been found in caves in Korea, but is described from soil in Japan. The identity of the Korean specimens and the original specimens from Japan may be questioned given the diversity of the genus (Yoshii 1995). No other location in cave habitats is mentioned in the literature for Allonychiurus, in contrast to Onychiurus which is highly diversified in caves. Two species (Allonychiurus pamirensis and Allonychiurus tianshanicus) live at high altitude in central Asia. Two others (Allonychiurus donjiensis and Allonychiurus jindoensis from Korea) are coastal halophilous species (Lee and Kim 1994); their generic assignation needs however confirmation, as this ecology and several morphological characters rather points to Thibaudichiurus from the same habitat and same region.

## Abbreviations and vocabulary used

### Material.

 The codes between brackets are field codes of the samples which contained the specimens, for instance (C9581).

### Material deposit.

 Nanjing University (China)—NJU, Museum national d’Histoire naturelle de Paris (France)—MNHN.

### Morphology.

Labial papillae types are named after Fjellberg (1999). Labium areas and chaetal nomenclature follow Massoud (1967) and D’Haese (2003). Chaetae on anal valves are named after Yoshii (1996).

Antantennal segments, AIIIOSensory organ of third antennal segment, PAOpostantennal organ, Ththoracic segments, Abdabdominal segments, p-chaetachaeta of row p on head, Spposterior S-chaeta (on Abd. V or on head), msS-microchaeta (microsensillum auct.), psopseudocelli, a-psopostero-internal pso on head, psppseudopore, ASanal spines, xaxial psp of Abd. IV.

The uneven axial chaeta m0 (Sun et al. 2010) of Abd. VI tergite is named here p0 in agreement with the literature.

Labral chaetae formula is the number of chaetae from prelabrals to distal row of labrum; for instance: 4/342.

Pseudocellar and pseudopore formulae are the number of pseudocelli and pseudopores by half-tergite (dorsally) or half-sternite (ventrally) as follows: head anterior, head posterior/Th. I, Th. II, Th. III/Abd. I, Abd. II, Abd. III, Abd. IV, Abd. V (for instance: 32/022/33343).

S-chaetae formula is the number of S-chaetae by half-tergite from head to Abd. VI (for instance: 11/012/222120).

Formula of tibiotarsal chaetotaxy: total number of chaetae (number of chaetae in the distal whorl (A+T), number of chaetae in the proximal whorl B, number of basal chaetae); for instance: 21 (11, 8, 2).

## Systematics

**Onychiuridae Börner, 1913**

**Allonychiurus Yoshii, 1995**

### Allonychiurus antennalis sp. n.

urn:lsid:zoobank.org:act:3F37A29C-FD87-45DD-94AC-C73FCFCF6A7C

Figs 1 2 Table 1. 

#### Type material:

Holotype female, 3 female paratypes. China: Jiangsu Province: Nanjing: Zijinshan: 10.iv.2009, litter, Berlese extraction, Zhang Feng et al. leg. (C9581). –ibid: Nanjing: Baima Park: 14 paratypes (2 males, 3 females and 9 juveniles) on slides, 13.v. 2007, litter, Berlese extraction, Chen Jian-xiu et al. leg. (C9544).

Holotype and 13 paratypes on slides are deposited in the Department of Biological Science and Technology of NJU, 4 paratypes on slides in MNHN.

#### Diagnosis:

pso formula as 32/133/33343 dorsally, 11/000/01000 ventrally; subcoxa 1 of legs I, II and III with 1, 1 and 1 pso respectively; parapseudocelli (psx) absent; presence of small, finely granulated, apical swelling at the apex of Ant. IV; Th. II and III each with 3 dorsal chaetae on both side of axial line; tibiotarsus with 11 chaetae in the distal whorl, no clavate tenent hair; ventral tube with 6+6 distal chaetae, without anterior or basal chaetae.

#### Description:

Body length: 1.3–1.7 mm (females), 1.0 mm (males). Body shape cylindrical, Abd. III–IV more or less broadened. Body colour white in alcohol.

**Figure 1.**
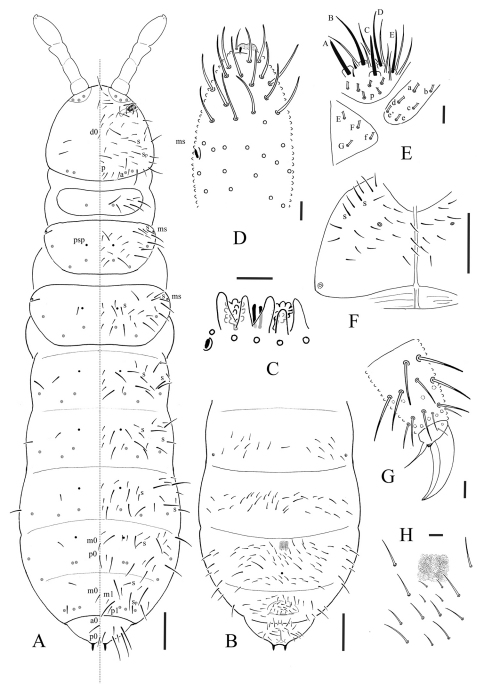
Allonychiurus antennalis sp. n. **A** dorsal side of body with chaetotaxy, S-chaetae, pso and psp **B** ventral side of Abd. II–VI **C** organ of Ant. III **D** dorsal side of left Ant. IV **E** labium (p, proximal group of chaetae of labial palp) **F** ventral side of head **G** distal part of leg III **H** furcal area. Scales: 0.1 mm (A, B & F), 0.01 mm (C–E & G–H).

Pseudocellar formulae as 32/133/33343 dorsally, 11/000/01000 ventrally (Figs 1A, B), subcoxa 1 of legs I, II and III with 1, 1 and 1 pso respectively. Parapseudocelli absent. Pseudopore formulae as 00/011/11110 dorsally, 00/111/000x0 ventrally (Figs 1A, B).

S-chaetae formula as 11/012/222120 dorsally. Sp present on head. S-microchaetae tiny and blunt, present on Th. II and III dorsally (Fig. 1A).

##### Head.

Antennae short and distinctly segmented, as long as head (Fig. 1A). Length ratio of antennal segments I: II: III: IV = 1: 1.8–2: 1.8–2: 3.8–4.0. Ant. I with 9–10 chaetae. Ant. II with 14–15 chaetae. Ant. III sensory organ composed of 5 papillae, 5 guard chaetae, 2 small rods and 2 weakly granulated sensory clubs, both morel-like; lateral ms just posterior to sensory organ (Fig. 1C). Ant. IV subapical organite rod-like; basolateral ms at about 2/5 length from base; presence of a small, finely granulated (probably only primary granulation), flat apical swelling at the apex of antenna (possibly remnant of apical bulb fused to the apex) (Fig. 1D); invaginated apical bulb absent. Antennal base with distinct granulation. PAO composed of 18–22 compound vesicles arranged in 2 rows along axis of organ (Fig. 1A). Dorsal cephalic chaeta d0 present. 4+4 p-chaetae between posterior a-pso on head (Fig. 1A). Mandible with strong molar plate and 4 apical teeth. Maxilla bearing 3 teeth and 6 lamellae. Maxillary palp simple with 1 basal chaeta and 2 sublobal hairs. Labral chaetae formula 4/342. Labium with 6 proximal, 4 basomedian (E, F, G, and f) and 6 basolateral (a, b, c, d, e, e’) chaetae; labial papillae of AC type, papillae A–E respectively with 1, 3, 0, 3 and 3 guard chaetae (Fig. 1E). Postlabial chaetae 4+4 along ventral groove (Fig. 1F).

##### Body chaetotaxy.

Ordinary chaetae differentiated in meso- and macro-chaetae, ratio Sp: m1: p1 on Abd. V = 1: 0.6: 1.1 (Fig. 1A). Th. I with 7+7 chaetae dorsally. Three chaetae on both side of axial line and no uneven axial chaetae from Th. II to Abd. III tergites. Abd. IV tergite with two uneven axial chaetae (m0 and p0 ), Abd. V tergite with one uneven axial chaeta (m0), Abd. VI with two uneven axial chaetae (a0 and p0) (Fig. 1A). Th. I, II and III sternites with 0+0, 1+1 and 1+1 chaetae respectively.

##### Appendages.

**Figure 2.**
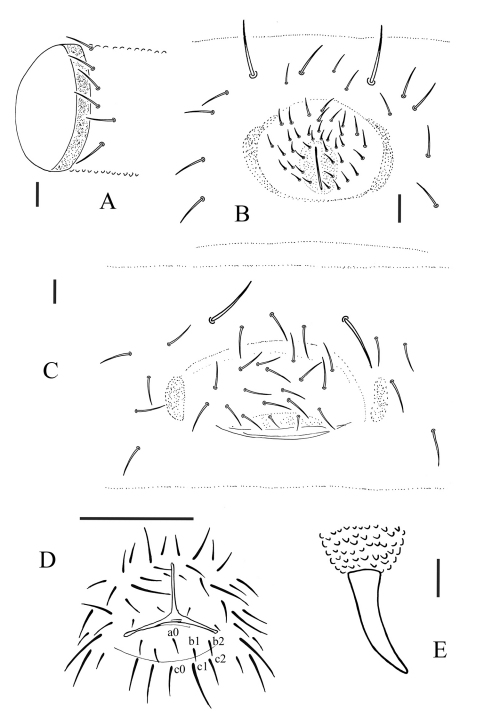
Allonychiurus antennalis sp. n. **A** ventral tube, lateral side **B** male genital plate **C** female genital plate **D** anal valves **E** anal spine. Scales: 0.1 mm (D), 0.01 mm (A–C & E).

Subcoxa 1 of legs I, II and III with 4, 4 and 4 chaetae, subcoxa 2 with 1, 4 and 4 chaetae, respectively. Tibiotarsi of legs I, II and III with 22 (11, 8, 3), 21 (11, 8, 2) and 21 (11, 8, 2) chaetae. Unguis without tooth. Unguiculus slender and pointed, 0.6 times as long as inner edge of unguis, with narrow inner basal lamella (Fig. 1G). Ventral tube with 6+6 distal chaetae, anterior and basal chaetae absent (Fig. 2A). Furca reduced to a finely granulated area, with 4 short chaetae in two rows posterior to furcal rudiment (Fig. 1H).

##### Male genital

plate with 30 circumgenital and 8 genital chaetae (Fig. 2B); female genital plate with 16–18 anterior and 2 genital chaetae (Fig. 2C). No modified chaetae ventrally in males. Anal valves with numerous acuminate chaetae; each lateral valve with chaetae a0 and 2 a1; upper (posterior) valve with chaetae a0, 2 b1, 2 b2, c0, 2 c1, 2 c2 (Fig. 2D). Anal spines set on distinct papillae, 0.6 times as long as inner edge of leg III unguis (Fig. 2E).

#### Derivatio nominis.

Named for its peculiar antennal morphology.

#### Ecology.

In broadleaved litter, in a recreational park in town, and at the forested foot of a hill close to the town, altitude 10 to 50 m a.s.l.

#### Remarks.

Bisexual species. Allonychiurus antennalis sp. n. can be easily recognized by the presence of its apical swelling on Ant. IV, not reported in other species of the genus. It has the same dorsal pseudocellar formula (32/133/33343) as Allonychiurus shinbugensis, Allonychiurus megasomus and Allonychiurus mediasetus. Diagnostic characters are summarized in Table 1.

**Table 1.** Comparison of the four species of Allonychiurus with a dorsal pseudocellar formula of 32/133/33343.

	Allonychiurus antennalis sp. n.	Allonychiurus mediasetus (Lee, 1974)	Allonychiurus megasomus Sun et al., 2009	Allonychiurus shinbugensis (Lee, 1974)
Ant. IV apical swelling	present	not mentioned	absent	not mentioned
Ventral pso formula	11/000/01000	11/000/01110	11/000/01110	10/000/01010
Inner basal lamella of unguiculus	present	present	absent	present
Number of chaetae on ventral tube	6+6	8+8	8+8	8+8
Number of chaetae on Th. I tergite	7+7	9+9	7-8+7-8	7+7
Number of p-chaetae between posterior a-pso on head	4+4	4+4	4+4	3+3
Number of axial chaetae on Th. II and III tergites	3+3	4+4	4+4	4+4
Uneven axial chaetae on Abd. IV	m0 and p0	p0	m0 and p0	m0 and p0
Maximum length (mm)	1.7	1.6	2.1	1.5

## Key to world species of Allonychiurus Yoshii, 1995

Note. Some forms of Allonychiurus flavescens from USA may lack pso on Th. I or are polymorphic (Christiansen and Bellinger, 1998). In the absence of more detailed information regarding other characters, they are not included in this key.

**Table:** 

1	AIIIO with 4 papillae, 2+2 or 3+3 pso on Abd IV	2
–	AIIIO with 5 papillae, more than 3+3 pso on Abd IV	3
2(1)	Dorsal pso formula 22/222/22222 after original description	Allonychiurus donjiensis(Lee & Kim)(South Korea)
–	Dorsal pso formula 32/233/33333	Allonychiurus jindoensis (Lee & Kim) (South Korea)
3(1)	Th. I tergite with 2+2 pso	4
–	Th. I tergite with 1+1 pso or without pso	5
4(3)	Th. I tergite with 12+12 chaetae, Abd. I tergite with 4+4 pso, dorsal pso formula 33/233/4444-54	Allonychiurus pamirensis(Martynova) (Russia)
–	Th. I tergite with 8+8 chaetae, Abd. I tergite with 3+3 pso, dorsal pso formula 32/233/34454 after original figure (given as 32/233/34445 in original description), ventral pso formula 1/000/00010, ventral tube with 8+8 distal chaetae	Allonychiurus tianshanicus (Martynova) (Russia)
5(3)	Th. I tergite without pso, dorsal pso formula 32/022/33343, ventral pso formula 11/000/00000, 4+4 p-chaetae between posterior a-pso on head, Th. I tergite with 8+8 chaetae, subcoxa 1 of legs I, II and III with 2, 2 and 2 pso respectively, ventral tube with 8+8 distal chaetae	Allonychiurus pseudocellitriadis (Lee) (South Korea)
–	Th. I tergite with 1+1 pso	6
6(5)	Th. II and III each with 3+3 pso dorsally; dorsal pso formula 32/133/33343	7
–	Number of pso on Th. II and III not as above	10
7(6)	Ventral pso formula 11/000/01110, 4+4 p-chaetae between posterior a-pso on head	8
–	Ventral pso formula not as above	9
8(7)	Distal whorl of tibiotarsi with 9 chaetae (interpretation of original drawing), Th. I tergite with 9+9 chaetae, unguiculus with basal lamella, Abd. IV tergite with one uneven axial chaeta (p0) dorsally	Allonychiurus mediasetus (Lee) (South and North Korea)
–	Distal whorl of tibiotarsi with 11 chaetae, Th. I tergite with 7-8+7-8 chaetae, unguiculus without basal lamella, Abd. IV tergite with two uneven axial chaetae (m0 and p0) dorsally	Allonychiurus megasomus Sun, Yan & Chen (China)
9(7)	Ventral pso formula 10/000/01010, Ant. IV without apical swelling, 3+3 p-chaetae between posterior a-pso on head, 4+4 axial chaetae on Th. II and Th. III tergites, ventral tube with 8+8 distal chaetae	Allonychiurus shinbugensis (Lee) (South and North Korea)
–	Ventral pso formula 11/000/01000, Ant. IV with a flat apical swelling, 4+4 p-chaetae between posterior a-pso on head, 3+3 axial chaetae on Th. II and Th. III tergites, ventral tube with 6+6 distal chaetae	Allonychiurus antennalis sp. n. (China)
10(6)	Th. II and III with 2+2 and 2+2 pso dorsally; dorsal pso formula 32/122/33343	11
–	Number of pso on Th. II and III not as above	13
11(10)	Unguiculus with basal lamella, Th. I tergite with 9+9 chaetae, Abd. V tergite without uneven axial chaeta dorsally after original drawing	Allonychiurus shanghaiensis (Rusek) (China)
–	Unguiculus without basal lamella, Th. I tergite with 8+8 chaetae, Abd. V tergite with one uneven axial chaeta (m0) dorsally	12
12(11)	Ventral pso formula 10/000/01000, Abd. IV tergite with two axial chaetae (m0 and p0) dorsally	Allonychiurus kimi (Lee) (South and North Korea)
–	Ventral pso formula 10/000/01110, Abd. IV tergite with one axial chaeta (p0) dorsally	A. flavescens (Kinoshita) (Japan, South Korea, USA)
13(10)	Dorsal pso formula 32/123/33343, distal whorl of tibiotarsi with less than 11 chaetae (interpretation of original figure)	Allonychiurus hangchowensis (Stach) (China)
–	Dorsal pso formula 32/132/33343, distal whorl of tibiotarsi with 11 chaetae, 4+4 p-chaetae between posterior a-pso on head, 3+3 axial chaetae on Th. II and Th. III tergites	Allonychiurus jongaksanensis (Weiner) (North Korea)

## Supplementary Material

XML Treatment for Allonychiurus antennalis
